# Communication, Social Networks and Sustainable Development Goals: A Reflection From the Perspective of Social Marketing and Happiness Management in the General Elections in Spain

**DOI:** 10.3389/fpsyg.2021.743361

**Published:** 2021-09-08

**Authors:** Araceli Galiano-Coronil, Gloria Jiménez-Marín, Rodrigo Elías Zambrano, Luis Bayardo Tobar-Pesántez

**Affiliations:** ^1^Marketing and Communication Department, University of Cadiz, Cádiz, Spain; ^2^Communication Department, University of Seville, Seville, Spain; ^3^Economy Department, University Politecnic Salesian of Ecuador, Cuenca, Ecuador

**Keywords:** communication, social marketing, sustainable development goals, policy, social media, happiness

## Abstract

The challenges imposed by the global development agenda imply reflecting on the role and contribution of political parties to development processes in the online environment. Social networks have been characterised as a part of the strategies of political campaigns, as it allows political leaders to establish bidirectional communication with citizens. In this context, the present study aims to empirically explore the leading Spanish political formations' publications from a social marketing perspective. In this way, it will be possible to verify how issues related to the Sustainable Development Goals (SDGs) are addressed. On the one hand, this requires elaborating the communication profiles of the main political parties presented to the Spanish General Elections from 2015 to 2019. On the other hand, to analyse whether social themes better discriminate or distinguish one political party from another. For this purpose, a methodology based on text mining, content analysis from a quantitative and qualitative approach, and simple correspondence analysis has been used. Finally, it should be noted that the results of this research show that there are differences between political parties according to the social issues published, with a divergence between the social issues that provoke a better reaction from the public and those most published on Facebook.

## Introduction

### Importance of Social Networks and the Application of Facebook in the Political Context

The so-called ICTs, Information and Communication Technologies, are part of the evolutionary framework of mass media and introduce qualitative changes in the social forms of interaction between people. This context entails a set of social, economic, political, and, above all, communicative manifestations that significantly impact how people interact with each other. Since the creation of the internet, and with the spread of the world wide web, interpersonal relationships have been digitised to the point that any citizen can express their opinion and actively organise around common interests thanks to the digital society (Porcelli, 2020) and the knowledge society (Silva et al., 2012). The society of the twenty-first century is a network society, that is, a society built around personal and corporate networks operated by digital networks that communicate through the internet. Moreover, since networks are global and know no boundaries, the network society is a society of global networks (Castells, 2012). The expansion of social networks worldwide is evident, as shown in different studies (Vepsäläinen et al., 2017; IAB Spain, 2021). Despite other growing networks, Facebook is still the social network with the most users in the world (Fernández, 2021).

To this, we must add that, in the last decade, social networks have been gaining importance as a communication tool used by companies (Marín Dueñas and Simancas, 2019) or by political parties (Pérez-Curiel et al., 2021). All this establishes a base where citizens can interact (Cabezuelo and Martínez, 2010), not only among themselves, but also through other media (González-Oñate et al., 2020) and where citizens and voters can be persuaded through political campaigns with propagandistic overtones (Gunn and Skogerbø, 2013; Bellido-Pérez and Donstrup, 2020).

Political communication has been transformed thanks to this multiplication and dissemination of messages through social networks, with efficiency and economic profitability rates that were unthinkable until now (Elías Zambrano et al., 2019). In this sense, a space for information, communication and propaganda opens up thanks to which political parties gain access to an online audience by invading their personal spaces (Bernhardt et al., 2012). Parties can now engage in dialogue with users (and voters) and address issues of interest since they can express their opinions (Levonian, 2016).

Currently, there are several studies on the use of Facebook in the political context, a large number of them for predictive purposes, such as Vepsäläinen et al. (2017), who examine the extent to which Facebook likes can be used to predict election results; and the research of David et al. (2016), who argue that it is desirable to identify the targeting of the potential electorate, as personalised messages are much more effective. Engagement is another issue that has been analysed on numerous occasions (Garz et al., 2020; Sally and Wickramasinghe, 2020). Another topic that has been of particular concern to researchers in this context has been the persuasion technics, the emotional tone and content of Facebook conversations, with a negative emotional connotation being the general tone (Bronstein, 2013; Abejón and Mayoral, 2017; Falck et al., 2020; Park et al., 2021; Widmann, 2021). Other authors approach this topic from the perspective of social network analysis, such as the research by Bíró et al. (2016). They explore whether the use of likes (on pages) and posting activity are relevant factors in network growth. However, the results show that parties form closed communities in the networks and have their users.

In Spain, some studies have investigated how users of the leading Spanish parties interact on Facebook. The results point to the existence of an interaction between users characterised by disagreement and more negative and offensive language, especially on the Facebook pages of the most ideologically polarised parties (Fenoll and Cano-Orón, 2017). Also, there are studies about misinformation (Cano-Orón et al., 2021), citizens' comments on Facebook about political leaders' messages (Zurutuza-Muñoz and Lilleker, 2018), or parties' engagement with citizens on Facebook (Ballesteros and Garrido, 2018).

In short, this proliferation in social media has led to a transformation in political communication in which political parties have seen these tools as essential resources that allow them to access an online audience. In this way, they open up space where users can express themselves on issues of topical interest (Levonian, 2016). Current issues include education, employment, welfare, equality, happiness, Etc., which have been gaining prominence in recent decades, especially since 2015, when the international authorities, which are part of the United Nations, met at the Sustainable Development Summit and drew up the 2030 Agenda with the Sustainable Development Goals (United Nations, 2021). Despite the importance of these social issues, insufficient evidence was found in the sources consulted on how political parties deal with the sustainable development goals in their Facebook communications, which will be addressed in the following section.

### Facebook, Social Marketing and Sustainability in the Political Context

Digital social networks (DSN), including Facebook, use the internet as a communication channel for socialising and sharing ideas. In these writings, the textual content of the DSN can be enriched with multimedia elements. Together with the interaction, immediacy, and public nature of the conversations, this characteristic makes these tools an excellent potential for the digital public sphere (Milliken and O'Donell, 2008). Moreover, chats generate relevant content for users (Bruns, 2006; Leong and Ho, 2020). As mentioned above, Kaplan and Haenlein (2010) define DSRs as.

“A group of internet applications based on the ideological and technological foundations of Web 2.0, which enable the creation and sharing of user-generated content” (p. 61).

If we consider the paragraphs above, it should be noted that social media has become a popular channel for political issues and opinions to exposure (Koiranen et al., 2020; Román-San-Miguel et al., 2021). According to Bond et al. (2012), social media can reflect citizens' political behaviour, which significantly influences their political decision-making. For this reason, politicians are increasingly using social media in their campaigns, as it allows political parties and organisations to connect with citizens in the age of the digital society (Strandberg, 2013).

Citizens' behaviour is a variable that can be influenced by the application of social marketing techniques in the political context. In this sense, Andreasen (1995) stated that social marketing aims to modify behaviours in search of a higher good (individual and social), being this change of attitude or behaviour, voluntarily, which has characterised this discipline in recent years. However, the paradigm of social marketing has evolved since the appearance and use of social networks as communication tools. In this sense, Hestres (2014) already suggested that, in a digital and social media context, efforts should be focused on getting the most significant number of users to talk about social issues, which would make it possible to offer engaging content to the public and, therefore, greater dissemination and visibility of the information. It would help leaders and political parties transform society (Bernhardt et al., 2012; Dooley et al., 2012).

However, despite the importance of issues affecting society such as poverty, health, equal opportunities, Etc. that are part of the SDGs, not much literature has been found that addresses these issues. Sustainable development meets the needs of the present without compromising the ability of future generations to meet their own needs (El Serafi, 1994). In this sense, at the United Nations Rio de Janeiro Conference on Sustainable Development (RIO+20) in 2012, 17 Sustainable Development Goals were developed to end poverty, protect the planet, and ensure peace and prosperity for people (United Nations, 2021). Some examples of research on sustainability focus on methodologies in different contexts such as education, such as the study by Lozano-Díaz and Fernández-Prados (2020). They proposed a research project on global and social citizenship under cyberactivism in university students of Education. This research showed a low level of active, critical and political engagement on the Internet about the environment, human rights or social justice in the online environment that would involve students' personal involvement. It was also observed that the learning methodology used, based on a workshop to train students in digital engagement issues in the field of the SDGs, resulted in a significant improvement in students' digital citizenship competencies. Another proposal focusing on using different methodologies for SDG analysis has been made by Ospina-Forero et al. (2020), who conducted a comparative study on the most suitable estimation methods for SDG networks, building a database with 87 development indicators in four countries over 20 years. The relationships between these development indicators (covering 16 of the 17 SDGs) during 1995–2004 for each country independently were analysed in this research. This information can be used for policy decisions.

Sustainability is also a goal that policymakers little discuss. An example is found in the issue related to climate change, where, while politicians may emphasise the urgency of addressing this issue, they often propose policies that weakly address the fundamental socio-economic roots of the problem (Chaffin et al., 2016). In this sense, numerous examples of policies are motivated by sustainability objectives, such as open space preservation strategies or people transit-oriented development (Krueger and Buckingham, 2012). These examples show that sustainability can remain a volatile notion, often ambiguously defined according to the political tactics of the parties involved in the decision-making process. It is reflected in the complete lack of public debate on sustainability-oriented forms towards objectives other than growth and progress (Latouche, 2009).

As a consequence, political debate remains limited to technicalities rather than political motivation. In other words, decision-making is increasingly seen as a matter of expertise rather than a political position. Political parties deliberately delegate issues to technicians to guide the process. It appears to be an intentional postponement of accountability, occurring as a defensive partisan strategy to cope with the risk of losing electoral power on issues of social relevance to the city (Bossuyt and Savini, 2018).

In short, according to most studies that have explored the relationship between the content of parties' and candidates' posts and the level of engagement they have generated on Facebook, they found that posts that talked about acknowledging their supporters or colleagues as well as those criticising their opponents perform better in terms of likes and comments than other types of content (i.e., information, mobilisation or individual posts) (Larsson and Kalsnes, 2014). Furthermore, regardless of ideology, political parties have made increasing rhetorical use of sustainable development over time, which, on the other hand, does not serve as a dimension for competition between political parties (Fleig and Tosun, 2017).

In this context, this study aims to empirically examine the publications of the leading Spanish political parties on Facebook from a social marketing perspective. In this way, it will be possible to verify how issues related to the Sustainable Development Goals (SDGs) are addressed. On the one hand, it is required for achieving this goal the elaboration of the communication profiles of the main political parties presented in the Spanish General Elections during the period between 2015 and 2019. On the other hand, to analyse whether there are issues that better discriminate or distinguish one political party from another. For this purpose, we have used a methodology based on data mining, content analysis from a quantitative and qualitative approach, and simple correspondence analysis. The findings of this work may have important practical implications for the future implementation of the happiness management philosophy by political parties. A culture that contributes to building the loyalty of political party voters through the intangible vector of happiness, together with assertive communication and supported by a political programme based on the fulfilment of the SDGs.

## Methodology

### Research Design and Study Sample

**Figure 1:**
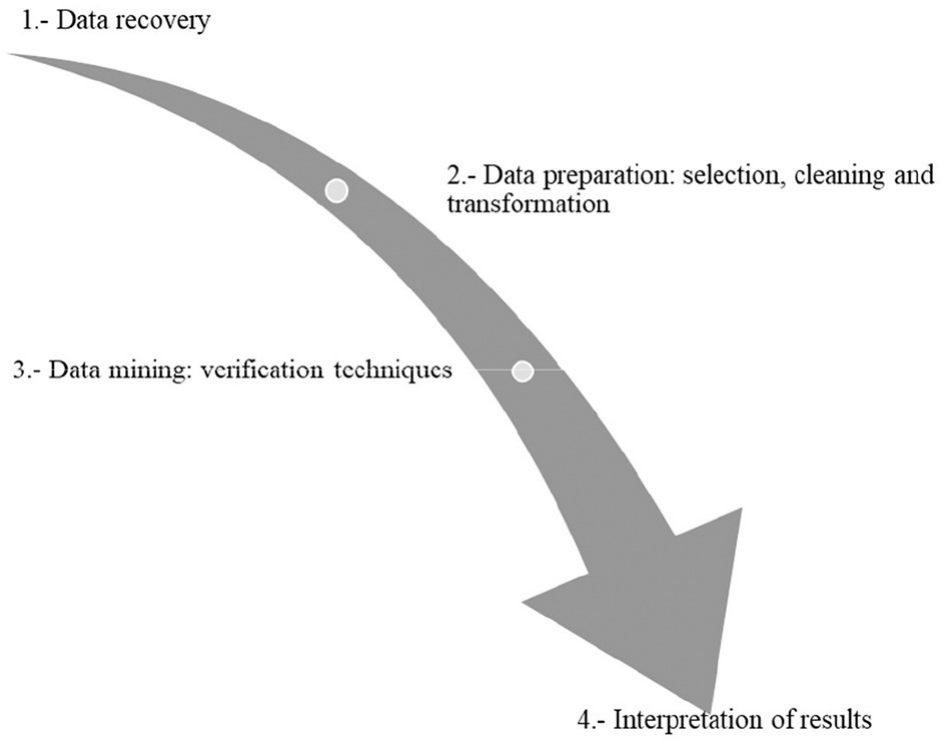
Phases of the KDD process. Source: Own elaboration based on Flores et al. (2019).

The specific characteristics of the data coming from social media (large volume of unstructured information) make it necessary to use a specific analysis methodology. This methodology must focus on extracting data and preparing and analysing to obtain valuable and relevant information that responds to the objectives. This procedure is known as Knowledge Discovery in Databases (KDD), defined by Maimon and Rokach (2010) as an organised process of valid, novel, helpful identification that generates understandable patterns from large and complex datasets. This process can be summarised in the phases specified in Figure 1.

Concerning the phases of the KDD process, it should be noted that data preparation (selection, exploration, cleaning and transformation) consists of eliminating unwanted messages, such as duplicate messages, and performing text cleaning by removing extraneous symbols, links, numbers, and numbers punctuation marks. Data mining allows us to discover new and potentially useful information from large amounts of data. Two types of techniques can be used; some are verification techniques, in which the system is limited to testing hypotheses provided by the user (for example, checking whether the use of videos influences the impact of social networks). Others are discovery techniques, in which potentially interesting patterns have to be found automatically, including all prediction techniques, for example, anticipating what kind of messages on social networks will have a more significant impact.

This study will be carried out using a descriptive approach to describe the particularities of the relevant groups and calculate the percentage of units showing a particular characteristic or determine the degree of association between variables (Malhotra, 2004). To this end, a content analysis was carried out (from both a qualitative and quantitative perspective) of the messages published by the main political parties in Spain: Partido Socialista Obrero Español (PSOE), Partido Popular (PP), Ciudadanos (Cs), Unidas Podemos (UP) and Vox. These political formations were chosen because they obtained the highest number of votes in the 2019 general elections, as specified in Table 1.

**Table 1:** Percentage of votes obtained by the most voted parties in the 2019 general elections.

**Political party**	**10th Nov 19**	**28th Apr 19**
PSOE	28	28.68
PP	20.82	16.7
VOX	15.09	10.26
UP	12.84	14.31
Cs	6.79	15.86

*Source: Own elaboration based on La Vanguardia (2019)*.

Posts published on Facebook by the selected parties during the selected periods were captured in an automated way using the Fanpage Karma tool. In total, 9,115 posts were collected. Of these posts, those mentioning social issues were analysed in detail, as explained in the section on content analysis, so that 2,965 posts were examined (Table 2), which were manually coded.

**Table 2:** No. of messages published by political parties discussing SDGs during General Elections from 2015 to 2019.

**Political parties**	**No. of messages published**	**No. of messages published about SDGs**
Cs	1,091	515
PP	3,000	778
PSOE	1,804	673
UP	1,345	364
VOX	1,875	635
Total	9,115	2,965

*Source: Own elaboration. Retrieved from https://www.facebook.com/*.

In addition, verification techniques have been used to test the following hypothesis tested:

H1: There are social issues (related to SDGs) that better discriminate or distinguish the different political parties.

To confirm the existence of a relationship between social issues and political parties, a simple correspondence analysis (SCA) was carried out.

### Content Analysis and Simple Correspondence Analysis

Content analysis is a technique that seeks to discover the significance of a message, whether it is a speech, a life story, a magazine article, Etc. More specifically, it is a method that consists of classifying and codifying the various elements of a message into categories in order to find patterns that give a specific meaning to the communication that takes place in them (Gómez, 2012). Krippendorff (1990) defined it as “A research technique designed to formulate, from certain data, reproducible and valid inferences that can be applied to their context” (p. 28).

The content analysis aims to achieve a quantitative information analysis and can study phenomena and data from a qualitative point of view. The first aims to measure the data and establish the frequency of occurrence of the elements considered units of information. The second (qualitative) allows us to verify themes, words or concepts in content. This research will take these two perspectives of content analysis to evaluate the effectiveness of the conversations published on the Facebook pages of the selected political parties.

For the procedure of this technique, the approach of Bardin (1983) will be used, which presents the following three stages: Pre-analysis, exploration of the material and processing of the results. Following these steps, we have identified as units of analysis the messages published on the Facebook pages of the political parties, PSOE, PP, Cs, UP and VOX, during the general elections of 2019, 2016 and 2015. The objective to be achieved with this technique is to elaborate the communication profiles of the political parties according to the social issues related to SDGs addressed by the publications. The following variables and categories have been identified to enable the coding of the messages:

- Likes: number of likes for each message.
- Comments: number of comments on each message.
- Political party: PSOE, PP, Cs, UP, VOX.
- Elections, according to the date of the General Elections: Election 1 (E1; 10th Nov 19), Election 2 (E2; 28th Apr 19), Election 3 (E3; 26th Jun 16) and Election 4 (E4; 20th Dec 15).
- Types of campaigns: Out-of-Campaign (OutC), Official Campaign (OC) and Pre-Campaign (PC), each of these types of campaigns are differentiated by the period that defines them. The PC corresponds to the period between the date of entry into force of the Royal Decree-Law calling the elections and the day before the start of the official campaign; the CO period is the period legally stipulated for the development of the electoral campaign, and the OC period is 1 month before the start of the PC period. Detailed information on the periods as mentioned above is specified in Table 3.
- Social issues. The categories of the social issues variable refer to the Sustainable Development Goals (SDGs) mentioned in the messages. These categories were identified by considering the wording of the SDGs according to the Ministry of Foreign Affairs, European Union and Cooperation (2015) and by examining the texts of the messages published by the political parties in the period considered. The most published words related to sustainable development issues were detected through this inductive process and are presented in Table 4.

**Table 3:** Campaigns corresponding to the Spanish General Elections under study.

**Elections**	**Election dates**	**Royal Decree-Law to dissolve the congress of deputies and the senate and calling for elections**	**B.O.E**	**PC period**	**OC period**	**OutC period**
E1	10th Nov 2019	RD 55/2019 24th Septermber 2019	230	24th Sep to 31st oct	01st Nov to 08th Nov	24th Ago to 23rd Sep
E2	28th Apr 2019	RD 129/2019 4th March 2019	55	04th Mar to 11th Apr	12th Apr to 26th Apr	03rd Feb to 03rd Mar
E3	26th Jun 2016	RD 184/2016 3rd May 2016	107	03rd May to 09th Jun	10th Jun to 24th Jun	02nd Apr to 02nd May
E4	20th Dec 2015	RD 977/2015 26th October 2015	257	26th Oct to 03rd Dec	04th Dec to 18th Dec	25th Sep to 25th Oct

*Source: Own elaboration*.

**Table 4:** Words identifying social issues.

**Social issues**	**Words**
Employment	Employment, young people, job, laboral, pensions, retirement
Gender	Iquality, gender, woman/en, girl, sexual, empowered, man/en, chauvinism, chauvinist, feminism, feminist, discrimination (in a gender context)
Environment	Deforestation, climate, carbon, emission, enviroment, worming, temperature, planet.
Health	Health, disease, illness, sick, health, donor, hospital, doctor, medicine
Poverty and exclusion	Poverty, water, hunger, disability, discrimination, drugs, poor, inequality, inequality.
Education	Teaching, education, teacher, school, culture, student, scholarship, university.
Economy	Economic, income, partnerships, cooperation, economy, taxation, rural, growth, consumption, trade, debt, production, depopulation.
National policy	Catalan, Barcelona, Torra, Catalonia, Generalitat, Parliament, Puig Demont, yellow ribbons, corruption.
Peace	Peace, terrorism, crime, dialogue, freedom, justice, crime, democracy, weapon, extremism, unity, coexistence, refugees.
Others	When terms are used in a message that refers to several social issues together.

*Source: Own elaboration*.

In order to determine the validity of these categories and decide whether they are the ones that will ultimately be used in the research, two experts in the field were consulted and provided with a sample of 100 publications chosen at random to be classified into the proposed categories. In this way, the concordance between the classifications of the two experts can be checked. Cohen's kappa coefficient was used to measure this, which yields a significance level of < 0.05. It indicates that a relationship is established between the two specialists. It indicates that the relationship between the two rankings is established, and the null hypothesis that there is no concordance between the results of the two experts is rejected. Also, the value of this parameter is 0.709, which indicates that the agreement between the two coders is satisfactory according to the scale of the researcher mentioned above (Warrens, 2014).

Once the coding process has been carried out, the next step is to deepen the knowledge of the messages. This process aims to extract the crucial questions on each parameter considered that support decision-making concerning social communication actions. For this purpose, the SPSS 25 plus programme has been used to carry out two types of analysis: a descriptive analysis and a correlational analysis. The latter aims to measure the degree of relationship between the parameters specified in the hypothesis to test.

In this study, SCA will confirm whether there is a relationship between political parties and the social issues addressed in Facebook posts. SCA is an interdependence technique that describes a data set and provides a perceptual map based on the association between objects and a set of descriptive characteristics or attributes (Hair, 1995). This multivariate technique first tests the association between variables using a chi-square test and then allows the latent relationship between the two variables to be identified graphically. The method starts with the construction of a contingency table of the variables analysed. Based on the information in this table, a two-dimensional perceptual map is constructed in which dots represent the categories of both variables. The closeness between the points associated with categories of different variables indicates the variables' level of dependence or association. In this sense, the various categories of the variables are represented closer or further away from the dimensions depending on their degree of similarity or differences (Zapillado, 2017). In other words, categories close to the origin of the two-dimensional space will be the categories of the variables that least discriminate each of the dimensions of the solution, and in the categories further away from the origin, the discrimination is more significant. Therefore, it is an ideal method for finding the underlying structure in sets of qualitative variables, with which we can work simultaneously and offer us the underlying behavioural patterns (Camacho, 2010).

## Results

### The General Communication Profiles of Political Parties

**Figure 2:**
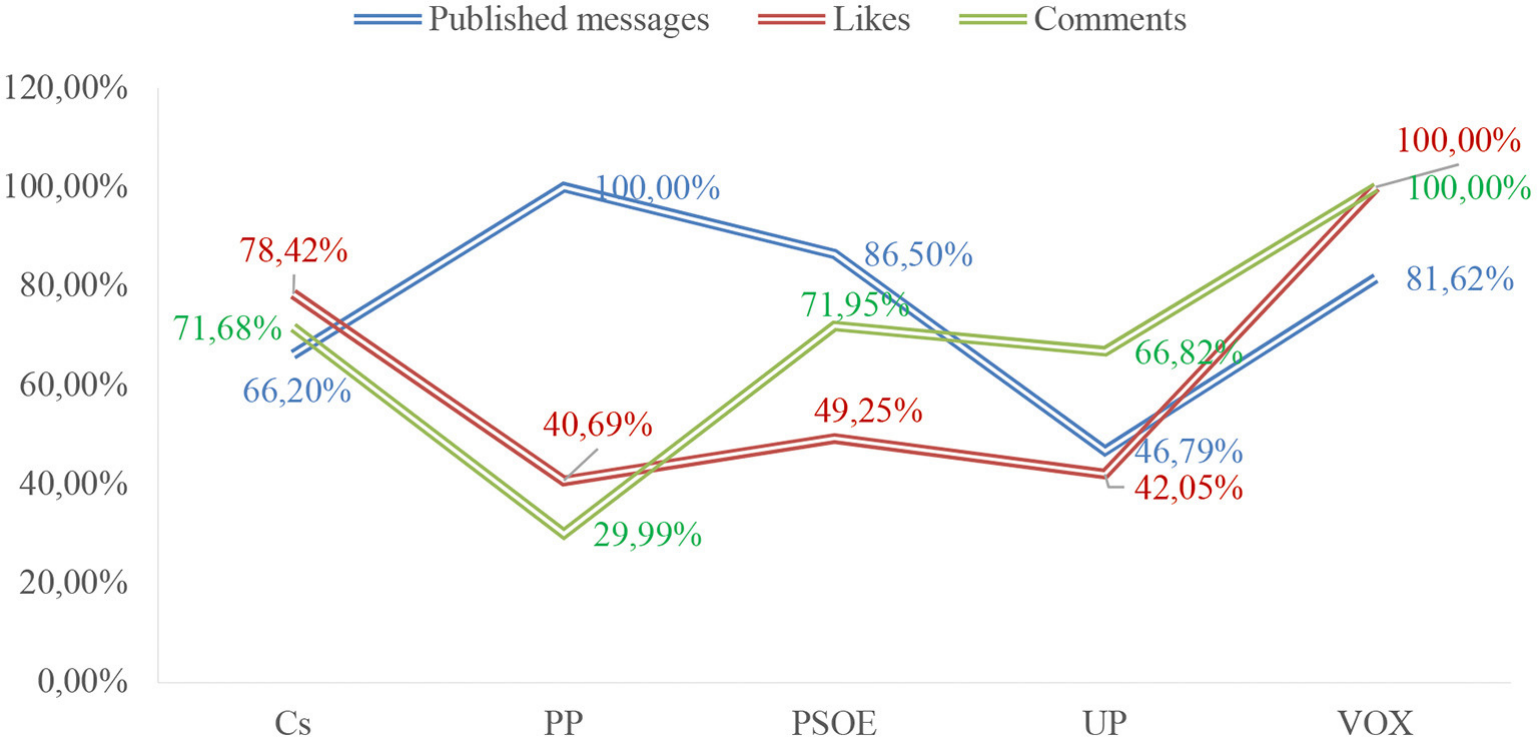
General communication profiles of political parties during the General Elections (2015-2019). Source: Own elaboration. Retrieved from https://www.facebook.com/.

Figure 2 below presents the general results of the descriptive analysis corresponding to the communication profiles of the political parties understudy during the 2015, 2016 and 2019 general elections. These results correspond to the analysis of the 2,965 messages selected for addressing social issues related to the SDGs.

Figure 2 shows that the most active parties in the period under consideration were PSOE, PP and VOX. Two interesting facts can be observed in this regard. The first is that Cs was the party with the second-highest average number of likes per message, despite being one of the parties that posted the fewest messages. Secondly, VOX was the party with the highest average number of likes and comments per message.

If we consider the activity corresponding to the different elections, Table 5 shows a disparity between the activity of the parties in the different elections and the public's reaction. More messages have been published in the April 2019 elections (E2), and the most active parties were Ciudadanos, PSOE and UP. However, the campaigns with the highest average number of likes per message were November 2019 (E1) and December 2015 (E4). As for the average number of comments per message, each party obtained the highest value for this indicator in a different campaign.

**Table 5:**
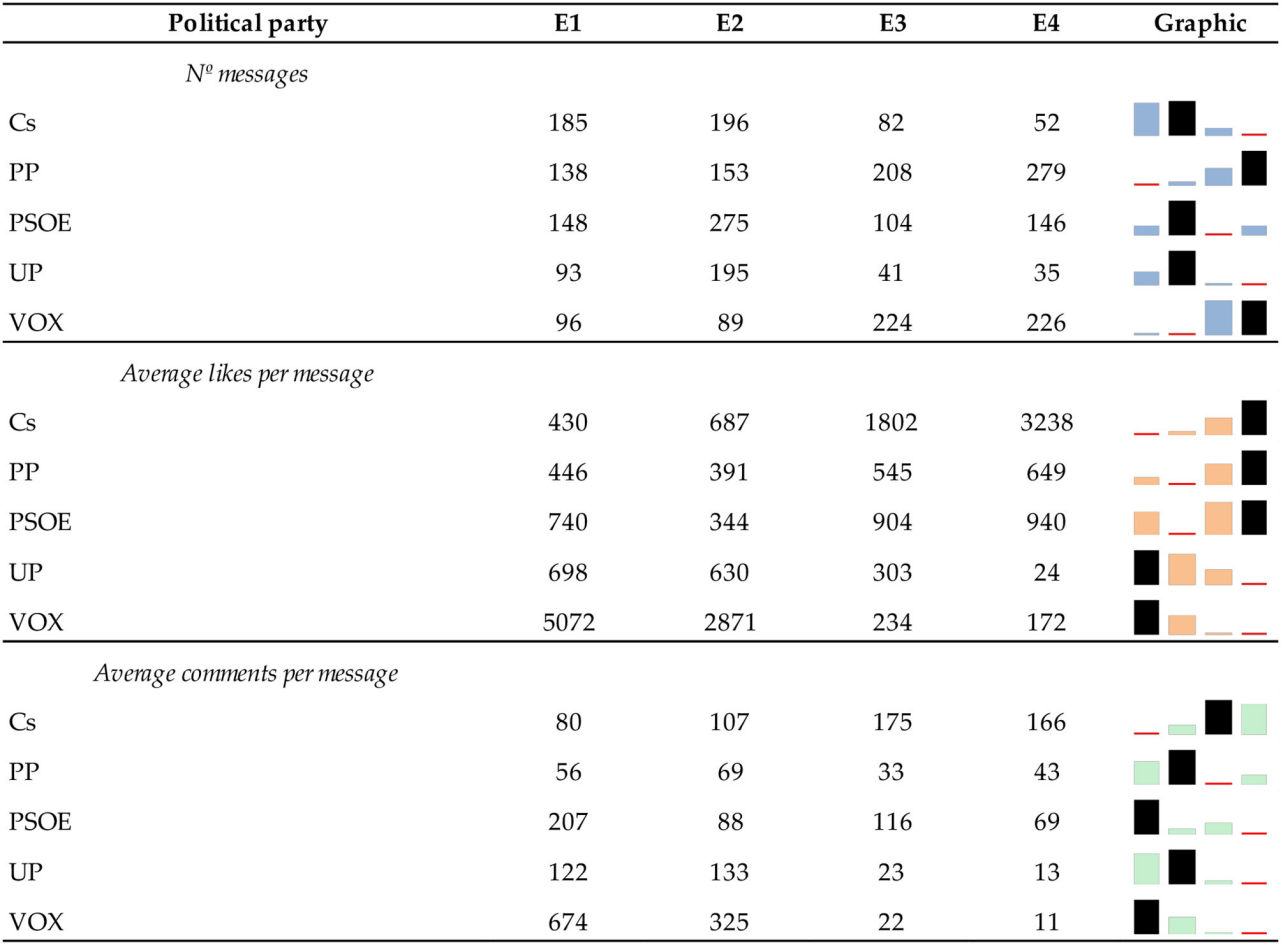
General communication profiles of political parties during the General Elections (2015–2019).

*Source: Own elaboration. Retrieved from https://www.facebook.com/*.

Table 6 below shows the messages of each political party that have obtained the most significant impact (measured by the sum of likes and comments):

**Table 6:** Original messages from each political party that have had the most number of likes and comments.

**Political party**	**Orginal message**	**N° likes + N° coments**	**General elections**	**Type of campaign**
Cs	?? ¿Te perdiste ayer a Inés Arrimadas diciendo las verdades a la cara al separatismo en el Parlament? !! ?? ”¡Es el señor Torra el que nos ha llamado bestias con forma humana!” ??! ‘No nos callarán! Mira ??	32.018	28th Apr 19	PC
PP	Conmocionados por los ataques ocurridos en la noche de hoy en París, condenamos de la forma más absoluta el terrorismo, venga de donde venga	6.382	20th Dec 2015	PC
PSOE	El PSOE quiere construir un país unido en el que haya igualdad de oportunidades.	9.713	20th Dec 2015	OC
UP	El viejo profesor Anguita es el político más honrado que hemos tenido, junto con José Antonio Labordeta. En España nos cuesta mucho elegir a los buenos porque siempre los medios de comunicación nos meten por los ojos a los malos. Nada sorprendente, ya que los propietarios de los medios de comunicación son los principales accionistas de las empresas del IBEX. Nunca van a poner la información al servicio del interés general, en este país, la información sirve a los intereses de la CASTA. Recuperemos este término porque el PSOE NO QUIERE DEJAR DE SER CASTA. Quique https://www.elmundo.es/papel/lideres/2016/02/21/56c6ef80ca474111668b4598.html?fbclid=IwAR2rDR7YGInG8y84yvCEmyJpjqGt3uTUHVvK2tDCUuGHLFjulxSzmC052V0.	5.104	10th Nov 2019	OC
VOX	? Santiago Abascal pulveriza a Pablo Iglesias “No me va a dar lecciones de democracia ni de defensa del orden constitucional. Yo me jugaba la vida amenazado por ETA en el País Vasco mientras usted presumía de que ETA tenerspicacia política”. ?? #ElDebate4N #DebateElectoral.	34.242	10th Nov 2019	OC

Table 6 shows that most messages talk about terrorism and national politics (Catalan separatism), related to occurrences close to the General Elections. In November 2015, Paris suffered several terrorist attacks (claimed by the jihadist organisation Islamic State) on terraces, bars, restaurants near the Stade de France, and the Bataclan concert hall. The attacks killed 130 people and injured 325 others (El Mundo, 2015). VOX also alludes to terrorism, referring to the nationalist terrorist organisation ETA (Euskadi Ta Askatasuna in Basque, Basque Country and Freedom), which perpetrated more than 800 murders in Spain (Ministerio del Interior (Gobierno de España), 2019). On the other hand, Catalan independence is another of the social issues with the most significant impact on the data analysed. However, based on the results obtained in Figure 1 and Tables 5, 6, it is worth asking what may have been the reasons for this disparity in the results. In order to answer this question, we will examine the messages from a qualitative and quantitative perspective, checking which social issues have been the most published social by each political party and which have encouraged a more significant reaction from the public.

### Analysis of Social Issues and Communication Profiles of Political Parties

**Figure 3:**
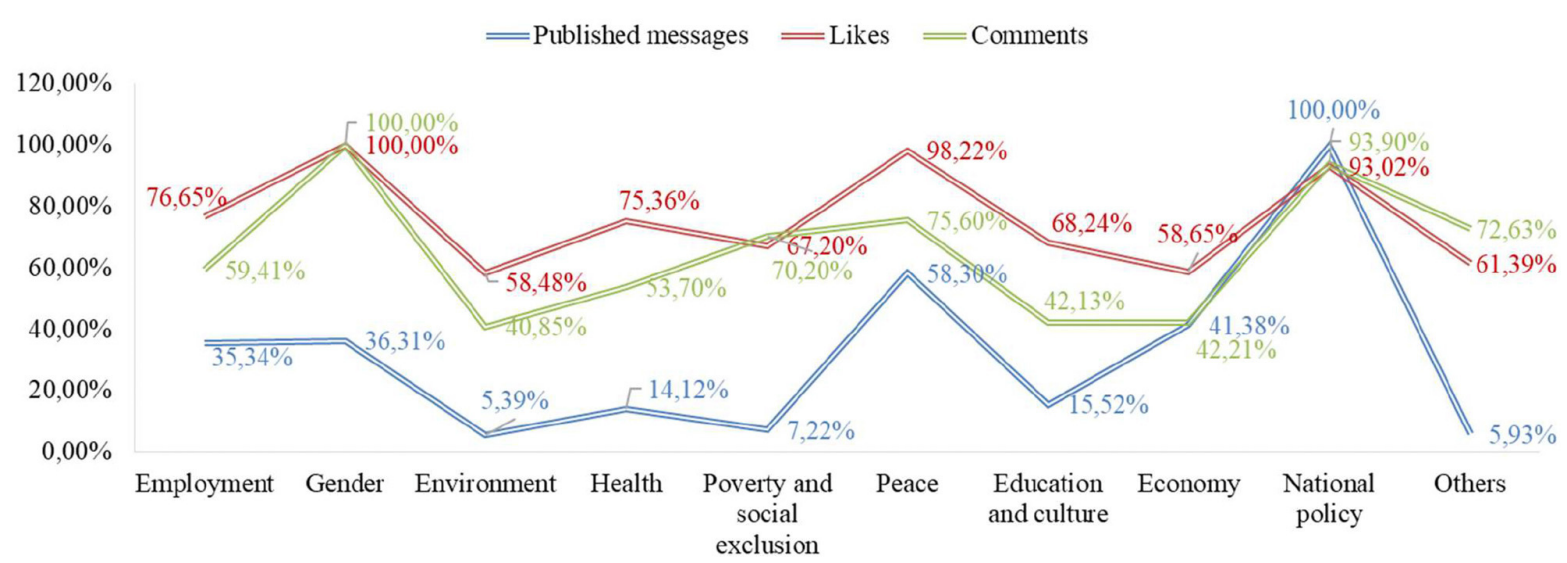
Communication profiles of political parties according to the social topic. Source: Own elaboration. Retrieved from https://www.facebook.com/.

Figure 3 shows the most published social topics and the average number of likes and comments per message according to each topic.

Figure 3 shows that the most recurrent social topic was national politics, which received the most likes per message. Examining the graph shows that the topic that has obtained the highest number of comments per message is gender. For a more detailed analysis, Tables 7, 8 show the communication profiles of each political party.

**Table 7:**
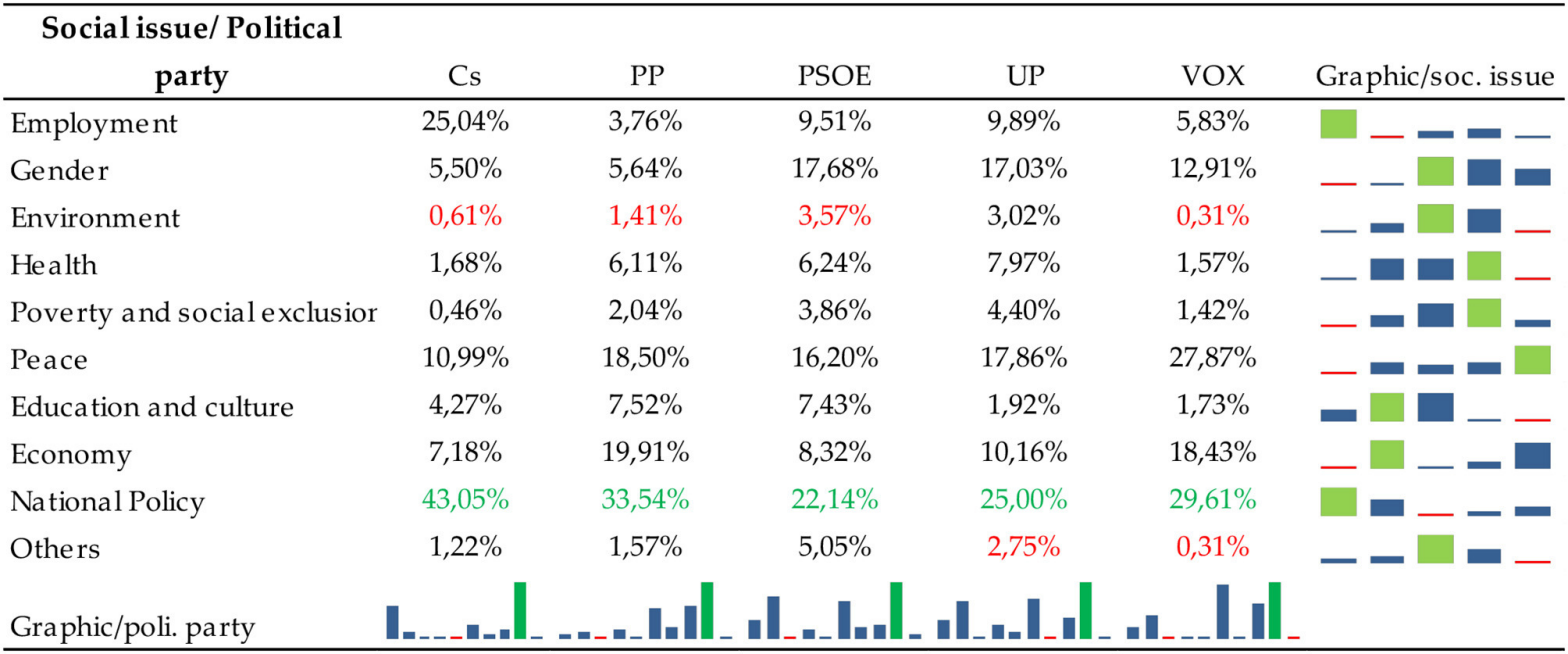
Social issues posted by political parties on Facebook during the 2015–2019 General Elections.

*Source: Own elaboration. Retrieved from https://www.facebook.com/*.

**Table 8:**
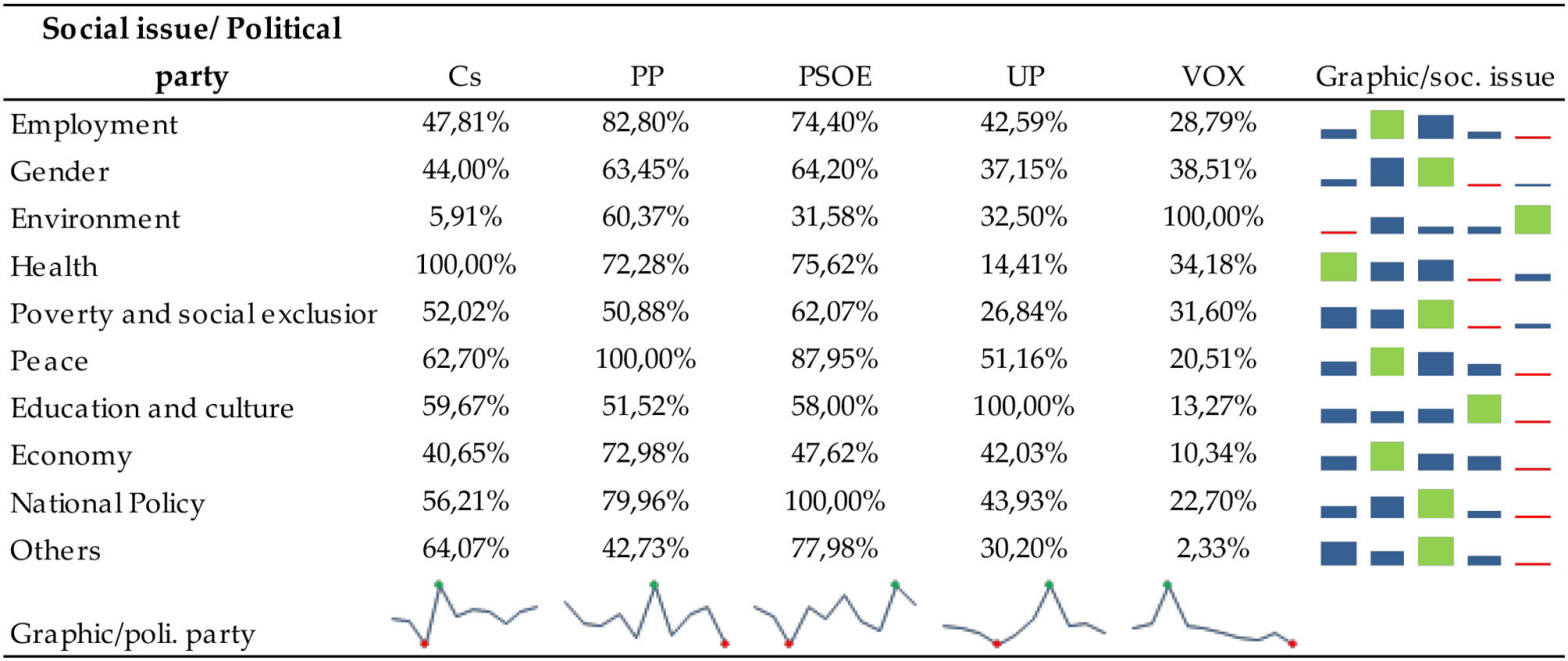
Impact (likes+comments) of messages that address social issues in each match.

*Source: Own elaboration. Retrieved from https://www.facebook.com/*.

Table 7 highlights several aspects: firstly, the least published social topic was the environment, and secondly, the most frequently published topic was national politics. Concerning national politics, it should be noted that the most frequent terms have been those related to the separatist issue or the independence of Catalonia and corruption. The separatist issue refers to the Catalan sovereignty process, known as the “Catalan process.” It refers to a set of social and political events developed since 2012 in Catalonia's autonomous community to achieve its independence from Spain. The 1st of October 2017 marked the most decisive week of the whole process. An unconstitutional referendum took place on that day, in a day with incidents with the police and in which the proper guarantees for a vote were not observed. On the 3rd of October, the King of Spain intervened publicly in defence of the constitutional order and the nation's unity. The European Union and member state governments unanimously took a clear stance against it, and thousands of companies moved their tax domiciles out of Catalonia. The week culminated in a massive demonstration in Barcelona against independence. Despite all this, the Parliament decided to go ahead with its plan: it approved the declaration of independence, which was instantly suspended, and gained no international recognition (Andreu, 2020). According to the Corruption Perceptions Index (Corruption Perceptions Index (CPI), 2019), corruption is another of the most worrying issues in Spain; it ranks 30th out of 180 countries surveyed in 2019.

A general trend was also observed concerning the most minor published topic, which was the environment. On the other hand, there is a disparity between the rest of the social issues. For example, in Cs, the second most published topic was work, PP economy, PSOE gender, UP gender and peace, and VOX peace.

Concerning public reaction, it is interesting to note two facts. On the one hand, there is a disparity between the social issues discussed by the different parties in terms of public impact (measured by the sum of likes and comments). That is to say, the Cs messages that have had the most significant impact have been those that talk about health, precisely one that talks about the Single Health Card to defend equality and to allow people to move freely and be attended to regardless of their place of residence. About the PP, messages about peace have had a more significant impact, with a message supporting Rajoy in response to aggression he suffered before delivering a speech at a meeting in A Coruña standing out. In the PSOE, messages on national politics are the most popular, such as advocating a government of change, which takes charge of the needs of citizens and undertakes public policy reforms that will make Spain a reference point for economic progress and social cohesion in the twenty-first century Europe. In UP, the reaction to a message that talks about Julio Anguita stands out, referring to his facet as a professor, so it would not be a message about education in the strict sense of the word. Finally, in VOX, the messages that stand out for their greater impact talk about the environment, especially one in which Iván Espinosa (deputy secretary of International Relations of the VOX political party) talks about the rural world. Once again, politics is once again taking the limelight away from social issues.

On the other hand, there are also differences between the issues most published by each party and those with the most significant impact. That is to say, the topics most posted by politicians are not the ones that have had the most remarkable public reaction. Table 7 shows that the most published topic in Facebook messages by political parties is national politics, marked by the separatist issue in Catalonia and corruption. However, the only political party that has had significantly impacted the messages that talk about this topic has been PSOE. In the others, the reaction has varied, and the issues that have provoked a more significant reaction from the public have been different in each party.

The mention above leads us to think that there may be an association between political parties and the different social issues they publish. In order to verify this, a simple correspondence analysis has been carried out, which will also allow us to create a perceptual map to determine the positioning of the political parties concerning the social issues they publish about and show the similarities or differences between the parties themselves.

Since correspondence analysis is only appropriate if the variables involved in the model show some degree of dependence, we first test this possible relationship between the publication of the various social issues and the political parties. The Chi-square test tests the null hypothesis that the variables considered are independent of each other against the alternative hypothesis that they are dependent. There is sufficient evidence to reject the null hypothesis for very low significance levels (<0.05) and therefore admit that the variables have some degree of relationship with each other. In Table 9, the significance level is null, so it is possible to admit a relationship between the various political parties and the social issues they post on Facebook. Table 9 also shows a complete solution with four dimensions or components, of which two together explain a proportion of inertia of 72.5%. These results make the application of correspondence analysis appropriate and establish a flat representation of the relationships between the variables that preserves 72.5% of the information.

**Table 9:** Summary table of the simple correspondence analysis.

**Summary**								
					**Proportion of inertia**		**Confidence singular value**	
**Dimension**	**Singular value**	**Inertia**	**Chi square**	**Sig**.	**Accounted for**	**Cumulative**	**Standard deviation**	**Correlation** **2**
1	0.259	0.067			0.392	0.392	0.017	0.061
2	0.239	0.057			0.332	0.725	0.019	
3	0.206	0.043			0.248	0.973		
4	0.068	0.005			0.027	1.000		
Total		0.171	513.186	0.000^a^	1.000	1.000		

a *36 degrees of freedom*.

**Figure 4:**
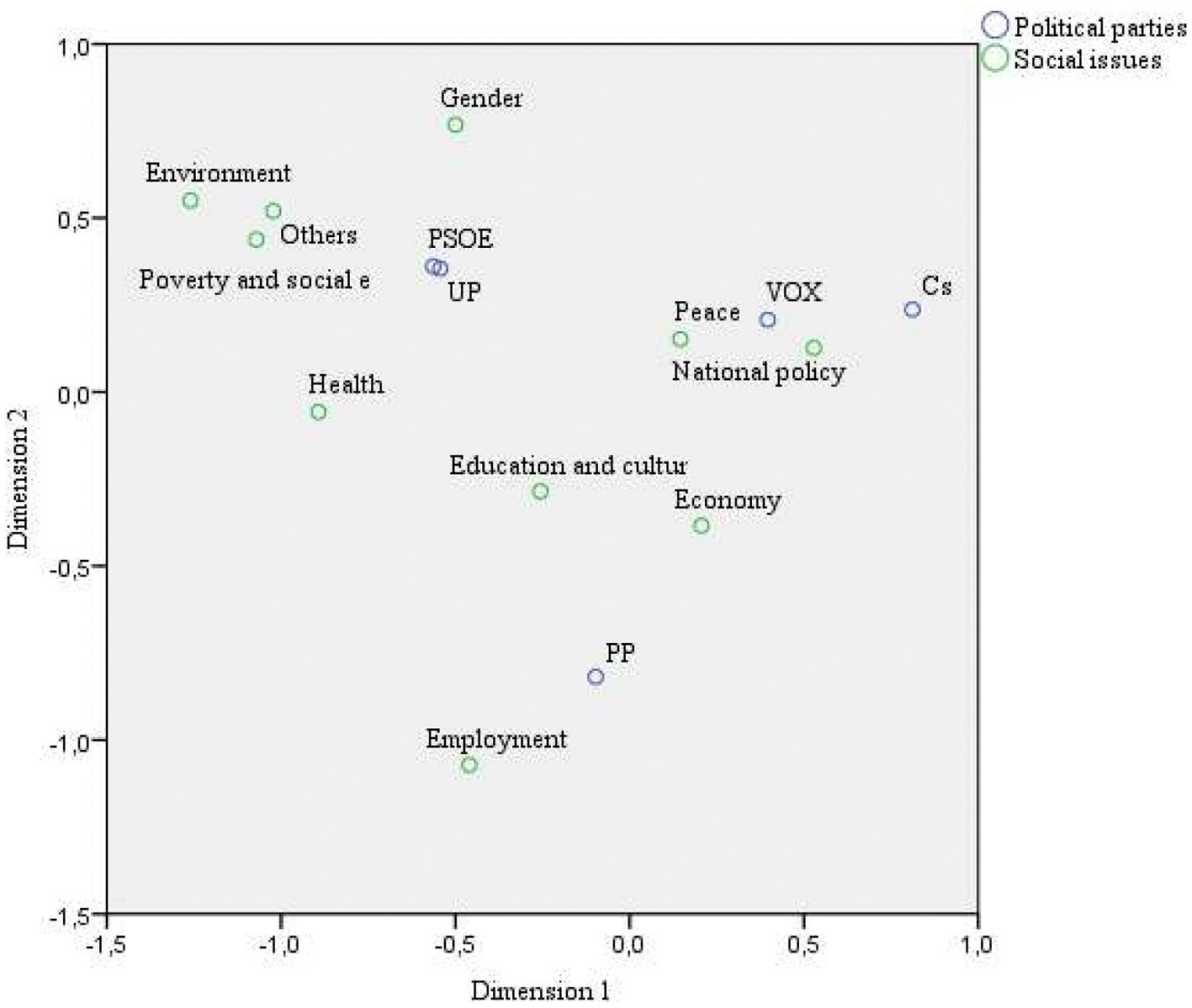
SCA Perceptual Map. Source: Own elaboration.

The perceptual map in the SCA determines a point on a Cartesian coordinate axis for each of the variable categories. The closeness of the points associated with different variable categories gives an idea of the degree of association between them. Based on the Figure 4, two exciting results can be concluded. On the one hand, there is a high association between the PSOE and UP parties, and between VOX and Cs (although the latter is not as strong), and on the other hand, concerning the possible associations between social issues and political parties, the following can be deduced:

There are issues that all political parties equally address, such as education and culture.

Other issues are more associated with some parties than others. In particular. There is a relationship between the themes of national politics and peace with the parties VOX and Cs, an association between the themes of environment, gender and health with PSOE, and a relationship between employment with PP. Therefore, we can conclude that social issues discriminate or better distinguish the different political parties, accepting the proposed hypothesis.

It should also be remembered that the SCA uses the Chi-square metric to measure the similarity between the different profiles. In this metric, modalities with low frequencies are weighted more favourably. It leads to the categories associated with low frequencies being located in extreme positions on the map. In this sense, it is noteworthy that the environment is positioned at one of the extremes, as is the Cs party, indicating the low impact of these categories in the study. In conclusion, topics such as the environment, employment and gender, although they still have some correspondence with certain political parties, are losing their discriminatory power in the context of conversations on Facebook on topics related to the SDGs.

## Discussion and Conclusions

This study aimed to explore the communication that the main political parties in Spain have carried out on Facebook during the General Elections from 2015 to 2019 on social issues related to the SDGs. It also aims to elaborate the communication profiles of these political parties in Spain according to the social issues addressed in their Facebook posts during the period under consideration. Concerning this objective, a hypothesis was put forward to test whether there is a relationship between the various political parties and the social issues published in their Facebook messages.

First of all, it is worth noting that political parties have been quite active on Facebook, using this social media as a tool in their communication campaigns. It is a fact that coincides with the majority of studies that have analysed social networks in the political context (Larsson and Kalsnes, 2014; Sorensen, 2016; Martinović et al., 2020; Rafałowski, 2020; Viounnikoff-Benet et al., 2020). Another fact to note is that the least active party has been UP, one of the newest political parties in Spain. We are agreed with Fenoll and Cano-Orón (2017) that citizen participation may have been influenced by a generational perspective, with younger supporters in the new parties and older ones in the traditional parties (CIS, 2016). Although it is true that, although the messages talk about social issues, it does not seem that these are the protagonists of the communication, but rather how they express the subject matter in the posts, which rather than inviting reflection on the importance of these issues try to mobilise voters or get their votes and compete with the opposition parties, which coincides with research such as that of Stetka et al. (2019) and Rafałowski (2020) focused on the Czech Republic and Poland.

According to the general communication profiles obtained, there is a disparity between the number of messages posted and the public reaction. That is to say, the parties that publish the most on social issues are not the ones that have achieved the most significant average impact in their messages (likes and comments). Thus, the PP stands out as the most active party, but the one that has achieved the most remarkable public reaction has been VOX. Considering the different General Elections held between 2015 and 2019, it should be noted that three of the five parties analysed, Cs, PSOE and UP, posted the most messages in the April 2019 elections, while PP and VOX posted the most posts in the 2015 elections. However, the only political party that has achieved the highest average number of likes in the elections where it was the most active has been PP. In the rest of the parties, there has been a disparity between the elections where they have posted more and achieved a more significant reaction from the public. Cs, PP, and PSOE have had the highest average number of likes in the 2015 elections, while UP and VOX in the November 2019 elections.

The results discussed in the previous paragraph led to the question of which social issues and under what circumstances the messages that have achieved the most significant public impact were posted. To this end, the communication profiles of the political parties were drawn up according to the social issues addressed in their Facebook messages, reaching the following conclusions: in general, the two most published social issues were national politics and peace, issues related to some of the main problems in Spain, the separatist movement in Catalonia, terrorism, corruption and refugees. However, the social issue with the most outstanding public response has been gender. The social and political debate on gender brought about by feminism and the homosexual and transsexual liberation movements is now a reality, which has fostered the importance of equal rights policies. In this sense, the results have shown that conversations on this issue have been effective, backed up by resources such as legal measures such as Royal Decree-Law 6/2019, of 1st March, on urgent measures to guarantee equal treatment and opportunities for women and men in employment and occupation.

In this sense, there is research that corroborates that, in the case of Spain, citizens have been active and committed to participating in local issues through Facebook and Twitter (Haro-de-Rosario et al., 2016; Fenoll and Cano-Orón, 2017).

It is noteworthy that the least published topic and the one that has received the minor public reaction has been the environment, even though attention to environmental issues is crucial due to its deterioration. As a result of these environmental changes, society becomes vulnerable to environmental and social hazards that have lasting impacts on human lives (Durán-Romero et al., 2020). We agree with the research conducted in Stockholm and Amsterdam by Bossuyt and Savini (2018), in which they state that most politicians allegedly delegate environmental issues to technical actors and are not the protagonists of their conversations on Facebook.

As practical implications, this paper proposes a new approach for the automatic identification of both the impact and the social issue orientation of political party conversations on social networks. It suggests that the political tendencies of both parties and individuals can be identified without recourse to any personal labelled data (David et al., 2016). The results obtained provide valuable insights into the differences between political parties and how they address social issues related to the SDGs.

Overall, the results suggest that political parties' online postings on Facebook do not address the root causes of SDG-related issues in depth. If they did, this could influence society's opinion, and thus on their future voting behaviour and behaviour on social issues.

Another practical implication is that social media postings give smaller or newer parties more opportunities than parties that have been around for many years and have more resources. In particular, social media enables communication at lower costs and allows for more equal campaigning conditions (Jost et al., 2018).

The study shows that it is worth studying the content on SDG-related issues posted by political parties on social media, as it tends to show different patterns. It requires further explanatory work involving more empirical material to be subjected to relevant quantitative and qualitative assessments. Moreover, this research contributes to other studies that attempt to build a forecasting model based on social media and how citizens and politicians engage with social media. As social media has become an important part of most citizens' lives in the digital age, it is significant to explore its impact on different research contexts and use alternative research methods.

One of the limitations of this work is that the automated text analysis based on word selection may lead to the exclusion of other terms related to the SDGs, and therefore, the analysis of the conversations has to be carried out with caution. However, validation of the social issue categories has been carried out by applying Cohen's Kappa coefficient (Warrens, 2014).

It should also be noted that our data focus exclusively on the Spanish political spectrum, so the results cannot be directly generalised to the international level. Another limitation of this study is that the analysis has been carried out only on Facebook, so it would be necessary to include other social media such as Twitter, one of the main communication tools in politics (Bernardes, 2020; Buccoliero et al., 2020).

Finally, concerning the time frame considered, it should be borne in mind that the context of the elections may favour polarisation among users and make it difficult to use Facebook as a space for opinion and discussion with points of view other than those of the parties. It would be appropriate to analyze more extended periods to verify the possible influence of the electoral context on the impact and quality of citizen participation.

In light of the results obtained and considering the complexity of political processes, our study urges research on how politicians engage with the SDGs. The increasing availability of low-cost, large-scale online social network data means that research can efficiently be conducted on the ground. If we want to understand genuinely—and improve—our society, well-being, and the world around us, it would be interesting to conduct studies to identify which real-world behaviours related to the SDGs are likely to be influenced through the online environment. In other words, one area of future research could be to compare whether or not behavioural changes are produced by the use of social marketing on social networks. In this regard, it should be noted that the impact of online communication on actual behavioural change outcomes is a complex area to measure and one that lacks academically published research studies (Thackeray and Neiger, 2009; Bennett, 2012).

In addtion, an analysis of the commentary on SDG messages could also be carried out.

In summary, the results of this study show a pattern that could be useful for future cross-national studies where social media use is common and political dynamics have evolved at an accelerated pace. It requires more detailed surveys and more specific research methods, such as social network analysis and qualitative analysis. In this sense, the research could also take a more qualitative approach by employing in-depth interviews to understand why the identified behaviour patterns are followed.

Therefore, it is argued that Facebook offers fertile ground for understanding political dynamics and suggests that this research offers starting points for pursuing a research agenda to help understand how users interact with political parties' messages on social issues (Galiano and Ortega, 2019).

## Data Availability Statement

The raw data supporting the conclusions of this article will be made available by the authors, without undue reservation.

## Author's Note

This article has been written during a research stay at the Faculty of Communication of the University of Seville in 2021.

## Author Contributions

AG-C, GJ-M, RE, and LT-P: conceptualisation, methodology, validation, formal analysis, investigation, resources, writing—original draft preparation, writing—review and editing, and supervision. AG-C: software and data curation. All authors have read and agreed to the published version of the manuscript.

## Conflict of Interest

The authors declare that the research was conducted in the absence of any commercial or financial relationships that could be construed as a potential conflict of interest.

## Publisher's Note

All claims expressed in this article are solely those of the authors and do not necessarily represent those of their affiliated organizations, or those of the publisher, the editors and the reviewers. Any product that may be evaluated in this article, or claim that may be made by its manufacturer, is not guaranteed or endorsed by the publisher.
